# Timing and reasons for coming late for the first antenatal care visit by pregnant women at Mulago hospital, Kampala Uganda

**DOI:** 10.1186/1471-2393-13-121

**Published:** 2013-05-25

**Authors:** Ivan Kisuule, Dan K Kaye, Florence Najjuka, Stephen K Ssematimba, Anita Arinda, Gloria Nakitende, Lawrence Otim

**Affiliations:** 1 School of Medicine, College of Health Sciences, Makerere University, Kampala, Uganda; 2 Department of Obstetrics and Gynecology, College of Health Sciences, Makerere University, Kampala, Uganda; 3 Department of Medical microbiology, College of Health Sciences, Makerere University, Kampala, Uganda

**Keywords:** Timing of first antenatal visit, Antenatal care, Mulago Hospital

## Abstract

**Background:**

Mothers who attend antenatal care late miss the opportunity of early detection of HIV and STDs, malaria and anaemia prophylaxis, health education and treatment or prevention of complications. Whereas many women in Mulago hospital make their first antenatal care visit after 20 weeks of gestation, the reasons for coming late are not documented. The objectives were to determine the gestation age at which pregnant women make their first antenatal care visit and the reasons for late coming.

**Method:**

The study was conducted in June 2012 among women with a gestation age of more than 20 weeks on their first antenatal care visit. We collected data on gestation age (from weeks of amenorrhea or based on ultrasound scan) and reasons for coming late.

**Results:**

Four hundred women participated in the study. Their mean age was 25.2 years with a standard deviation of 5.2 years. The majority of the participants were Catholics (n = 126, 31.5%), they lived in a distance of greater than five kilometers from the hospital (n = 201, 50.3%) and had attained secondary education (n = 220, 55.0%). The mean of their weeks of amenorrhea was 27.9 (± 4.6) weeks. The results showed that 291 (72.7%) of the study participants did not know the right gestation age at which a pregnant woman should start attending antenatal care. One hundred and ten (27.5%) agreed that they did not have money for transport to bring them to the hospital while 37 (9.3%) thought that they had to pay for the antenatal care services. Two hundred thirteen (53.3%) reported that they did not have any problem with their current pregnancy and so they saw no reason to come early for antenatal care, even though some of these knew the right gestation age at which they should make their first antenatal care visit.

**Conclusion:**

Pregnant women who come late for antenatal care in Mulago hospital, Uganda are not well-informed about the right gestation age at which they should make their first antenatal care visit and/or of the importance of early attendance at antenatal care.

## Background

Many health problems in pregnant women can be prevented, detected and treated by trained health workers during antenatal care visits. The World Health Organization (WHO) recommends a minimum of four antenatal visits, comprising interventions such as tetanus toxoid vaccination, screening and treatment for infections, and identification of warning signs during pregnancy [1]. The main objectives of antenatal care are: prevention and treatment of any complications; emergency preparedness; birth planning; satisfying any unmet nutritional, social, emotional and physical needs of pregnant woman; provision of patient education, including successful care and nutrition of the newborn; identification of high risk pregnancy; encouragement of (male) partner involvement in antenatal care [2]. The first of such antenatal visits should be conducted in the first trimester (before 14 weeks of gestation) [1]. Identification of complications or risk factors for complications on such early visits enables early institution of interventions to alleviate or mitigate the effects of such complications on the mothers and unborn babies [2].

Research has indicated that antenatal education for expectant mothers results in sustained improvement in knowledge of newborn care [3]. Another study done in Enugu, Nigeria showed that the prevalence of anaemia in pregnancy at booking was high (40.4%) and recommended that early antenatal booking and improved antenatal care are necessary for early diagnosis and treatment of the condition [4]. These studies emphasize the importance of early antenatal care in insuring a good outcome for both the mother and her baby.

Several factors affecting the utilization of antenatal care in developing countries have been identified [5]. These include: maternal education, husband's education, marital status, availability, cost, household income, women's employment, media exposure and having a history of obstetric complications. From a systematic review of the literature on factors that affect utilization of antenatal care, mothers who are educated, and those whose husbands are educated, are more likely to utilize antenatal care. Availability, affordability and easy accessibility of health units where antenatal care is offered increase utilization of antenatal care. Cultural beliefs and ideas about pregnancy also had an influence on antenatal care use, in that they may lead to mothers attending antenatal care late or not attending at all. Parity had a statistically significant negative effect on adequate attendance, where by women of a high parity tend not to attend antenatal care, attend late for the first antenatal visit or have few antenatal care visits. The quality of antenatal care might have an influence on utilization of antenatal care, leading to infrequent or late first visits to antenatal care. Whilst women of higher parity tend to use antenatal care less, this might be a result of an influence of women’s age or religious beliefs.

Globally, during the period 2000–2010, about 53% of pregnant women attended the recommended minimum four times antenatal care. The proportion of pregnant women in developing countries who attended at least one antenatal care visit has increased from approximately 64% in 1990 to about 81% in 2009 but, in low-income countries, only 39% of pregnant women attended four times or more antenatal care during 2000–2010 [1]. According to Uganda clinical guidelines of 2010, for normal (uncomplicated) pregnancies, four routine antenatal care visits are recommended as follows: the first visit between 10–20 weeks of pregnancy; the second visit between 20–28 weeks of pregnancy; the third antenatal care visit between 28–36 weeks and fourth antenatal care visit after 36 weeks [2]. The guidelines also recommend more frequent visits and early antenatal care visits for mothers with pregnancy complications, or those with identifiable risk factors for such complications, such as complications in a prior pregnancy [2].

The findings of the Uganda Demographic and Health survey 2011 showed that though over 90% of pregnant women attend antenatal care at least once, only 48% make four or more antenatal care visits during their entire pregnancy, only 21% of women made their first antenatal care visit before the fourth month of pregnancy, only 52% of women deliver under the care of a skilled birth attendant, and the maternal mortality ratio is 438 per 100,000 live births [6]. This implies that 79% of pregnant women come late for their first antenatal care visit. However, the actual gestation age at which they come and the reasons for coming late are not documented. The objectives of this study were to determine the time of gestation at which pregnant women who come late (at 20 weeks of gestation or beyond) make their first antenatal care visit and the reasons they give for this late coming to the first antenatal care visit. Knowledge of the information, particularly reasons behind coming late for the first antenatal care visit by some pregnant women, will enable development of recommendations to the concerned authorities (policy makers, health professionals and pregnant women) aimed at encouraging pregnant women to make their first antenatal care visit at the times recommended in the Uganda Ministry of Health guidelines.

## Methods

### Study site

The study was conducted in June 2012 in Mulago hospital which is the national referral and teaching hospital for Uganda. This hospital is located at Mulago hill, Kampala district in Uganda with a bed capacity of 1500 beds. This hospital has a directorate of obstetrics and gynecology which deals with women’s health. This directorate runs two antenatal clinics and has a maternity ward which conducts about 80 deliveries per day. Most of the women who attend antenatal care are low income women from the city, its suburbs and peri-urban areas, but there are also referrals from district and other lower level hospitals and private clinics. The antenatal clinic in upper Mulago was used as the study site. This clinic which targets low-risk women with no pregnancy complications or risk factors identifiable from history or examination runs from Monday to Friday. On average, the clinic receives100 pregnant mothers coming for the first antenatal visit per day.

### Study design

This was a cross sectional study where pregnant mothers attending the antenatal clinic for the first time with respect to the current pregnancy were recruited during the study period. The inclusion criterion was having a gestation age of more than 20 weeks with no prior history of antenatal care attendance in this pregnancy. Pregnant mothers presenting with more than 20 weeks of amenorrhea were considered to have come late in line with the recommendation by the Uganda clinical guidelines. For pregnant women who consented to participate in the study, interviews were conducted using a pre-tested interviewer-administered questionnaire. Clinical obstetric examination was conducted to determine the gestation age by estimation of the fundal height for all participants. The weeks of amenorrhea were calculated basing on the first day of the last normal menstrual period for those pregnant mothers who were sure of this date, after confirmation that they were not using contraceptives and their menses were regular prior to conception. We used an obstetric ultrasound scan to determine the gestational age at presentation for those pregnant mothers who were not sure of the date and those where there was a discrepancy between the weeks of amenorrhea and the fundal height gestation age.

### Sample size

The sample size consisted of 400 pregnant women. This ultimately gave a reflection of the total number of pregnant mothers coming late for their first antenatal care visit. Every other mother who had presented for the first antenatal care visit with a gestation age of more than 20 weeks and consented to participate in the study was included with no exclusion criteria.

### Data collection

Trained interviewers interviewed the pregnant mothers using a pre-tested questionnaire on the days of data collection. The questionnaire collected information about: a) socio-demographic characteristics including age, religion, distance from hospital, level of education of the pregnant woman and her partner, their occupation and marital status; b) obstetric history of the pregnant woman including date of last normal menstrual history , weeks of amenorrhea, gravidity, history of abortion and history of any problem in current or previous pregnancy; c) reasons for coming late including whether they had sought any medical care somewhere before coming to Mulago hospital, who decides that they attend antenatal care, what they thought as the right gestation age at which they should start attending antenatal care and why they had come late for the first antenatal care visit. The interviews were conducted in a private and calm environment so as to ensure confidentiality.

### Data analysis and processing

Collected data was entered twice into a computer using Epi info version 2000 and analyzed using SPSS version 16.0 and Microsoft excel. Continuous data was presented as mean and standard deviation while non-continuous data was categorized and the percentage of each category was calculated.

### Ethical consideration

We got ethical approval from the Makerere University, College of Health sciences, School of Medicine Research Ethics Committee and permission from the head of the directorate of Obstetrics and Gynecology and the In-charge of the antenatal clinic. Written informed consent was obtained from the study participants.

## Results

### Socio-demographic characteristics of the study population

Four hundred pregnant women participated in the study and their socio-demographic characteristics are summarized in Table 1. Their mean age was 25.2 years with a standard deviation of 5.2 years. The majority of the participants were Catholics (n = 126, 31.5%); they lived in a distance of greater than five kilometers from the hospital, (n = 201, 50.3%); had attained secondary education (n = 220, 55.0%); were house wives (n = 194, 48.5%) and married or in stable relationships (n = 306, 76.5%). Their partners’ education level was also predominantly secondary education (n = 220, 55.0%) and they were mainly self-employed business men (n = 315, 78.7%).

**Table 1:** Socio-demographic characteristics of the study population

**Characteristic**	**Number (n = 400)**	**Percentage**
**Age groups**		
15–19	56	14.0
20–24	145	36.2
25–29	116	29.0
30–34	58	14.5
35–39	24	6.0
40–44	1	0.3
**Religion**		
Catholic	126	31.5
Protestant	99	24.7
Muslim	100	25.0
Born again	68	17.0
SDA	7	1.8
**Address**		
Less than 3 km	73	18.2
3-5 km	126	31.5
Greater than 5 km	201	50.3
**Level of education of the pregnant woman**		
No education	7	1.8
Primary	141	35.2
Secondary	220	55.0
Tertiary	32	8.0
**Level of education of the partner**		
No education	3	0.8
Primary	35	8.7
Secondary	220	55.0
Tertiary	64	16.0
I don’t know	78	19.5
**Occupation of the pregnant woman**		
House wife	194	48.5
Peasant	4	1.0
Business/Entrepreneur	174	43.5
Salaried/Waged	21	5.3
Student	7	1.7
**Occupation of the partner**		
Not employed	5	1.3
Peasant	2	0.5
Business/Entrepreneur	315	78.7
Salaried/Waged	75	18.7
Student	3	0.8
**Marital status of the pregnant woman**		
Married	306	76.5
Single	88	22.0
Widower	1	0.3
Separated	5	1.2

### Obstetric history of the study participants

Table 2 shows the obstetric history. Among the 400 pregnant women, 329 (82.2%) were sure of the date of the first day of their last normal menstrual period (LNMP); the mean of their weeks of amenorrhea (WOA) was 27.9 with a standard deviation of 4.6 weeks. Two hundred thirty three (58.2%) pregnant women were multi-gravid. A history of abortion was reported by 86 (21.5%) pregnant women of whom 73 (84.9%) had one previous abortion and 32 (37.2%) did not know the cause of the abortion. One hundred seventy four (43.5%) reported a history of at least one problem in the current or previous pregnancy and the most frequently reported problems were having one or more previous scar(s) due to different indications and lower abdominal pain.

**Table 2:** Obstetric history of the study participants

**Characteristic**	**Number**	**Percentage**
**Date of first day of LNMP**		
Not sure of date	71	17.8
Sure of date	329	82.2
**WOA groups**		
20-28	224	56.0
29-36	160	40.0
Greater than 36	16	4.0
**Gravidity**		
Prime gravid	97	24.3
Multi gravid	233	58.2
Grand multi gravid	70	17.5
**History of abortion**		
Yes	86	21.5
No	314	78.5
**Number of abortions**		
One	73	84.9
Two	7	8.1
Three	4	4.6
Five	1	1.2
Seven	1	1.2
**History of at least one problem in current or previous pregnancy**		
Yes	174	43.5
No	226	56.5

### Reasons for coming late for the first antenatal care visit

Of the 400 pregnant women, 120 (30.0%) had sought antenatal care from other health facilities before coming to Mulago hospital and these were mainly health centers (n = 74, 61.7%), the other places being private clinics (n = 23, 19.2%), another hospital (n = 21, 17.5%) and from a traditional birth attendant (n = 2, 1.2%). Fifty five (45.8%) of these 120 women had been referred due to various reasons like being a prime gravid, being grand multi gravid, having a previous scar, twin pregnancy, pre-eclampsia, for PMTCT services etc. Forty (33.3%) of them had come to Mulago hospital because they want to deliver from there and so they wanted to get a file and book for delivery while 25 (20.8%) reported that they had shifted from one place to another and so Mulago hospital was nearer to their new residence.

When the study participants were asked that who decides that they seek antenatal care, 162 (40.5%) said it was them and their partner together, 140 (35.0%) took the decision alone, while the decision was made by their partner alone for 57 (14.3%) of the women. However, there were other people that were also reported by 41 (10.3%) of the women to influence this decision and these where the woman’s mother, father, aunt, grandmother, sister and friend.

The results showed that 291 (72.7%) of the study participants did not know the right gestation age at which a pregnant woman should start attending antenatal care. The 109 (27.3%) women who knew the right gestation age stated this time as three months (n = 83, 76.1%); four months (n = 17, 15.6%); two months (n = 6, 5.5%) and when a menstrual period is missed (n = 3, 2.8%). The majority (n = 101, 92.7%) of these109 women said that they were taught this time during health education in a health facility when they had attended antenatal care in previous pregnancies. Others said they had been told by their mother, sister or friend while one mother said she knew it from common sense. Still one mother said that she was taught in school.

One hundred ten (27.5%) of the study participants agreed that they did not have money for transport to bring them to the hospital while 37 (9.3%) thought that they had to pay for the antenatal care services. Two hundred thirteen (53.3%) reported that they did not have any problem with their current pregnancy and so they saw no reason to come early for antenatal care, even though some of these knew the right gestation age at which they should make their first antenatal care visit. Other varying reasons for coming late were given by 180 pregnant women (45.0%). These are summarized in Table 3.

**Table 3:** Other reasons given by the pregnant mothers for coming late

**Reason**	**Number**	**Percentage (n = 180)**
Was getting ANC from somewhere else	96	53.3
Was busy	24	13.3
Developed a problem	13	7.2
Was far away	12	6.7
Was lazy	9	5.0
The pregnancy has grown	8	4.4
Wants to get a file	6	3.3
Was told to come	5	2.8
Did not know where the ANC clinic is located	3	1.7
She has had children before	1	0.6
Doctors and nurses don’t pay attention when early	1	0.6
Got tired of ANC during past pregnancies	1	0.6
Thought this was the right time to come	1	0.6

## Discussion

This study showed that many pregnant women do not know the right time of gestation at which they should start seeking antenatal care which partly explains why they reported late for the first antenatal care visit. Not knowing the right gestation age at which to start the first antenatal care visit was the commonest reason for late attendance. The few pregnant mothers who knew the right time of gestation at which they should come for the first visit had been taught in health facilities during health education in previous attendance for antenatal care. This means that it is less effective to give this health education only during antenatal care attendance because the mother will already be late and worse still she may also come late on that particular day when health education has already been done. Further still, a prime gravid who has never attended antenatal care before will not know this information. In addition, there are other people who influence a pregnant woman to attend antenatal care and once these people are also not informed then pregnant women will not make informed decisions.

The average gestation age at first attendance for the pregnant women who attended their first antenatal care visit late was 27.9 weeks (approximately 7 months). This was higher than the mean gestation age at first presentation of 5 months reported by a study done in Kenya [7] and in South Africa [8]. However, these studies included even pregnant mothers who presented with less than 20 weeks of gestation. No other study was found to have studied late coming for first antenatal care visit or quality of antenatal care services.

The women in the study appear to have been of low socioeconomic status. This is evidenced by the fact that majority of the study participants had only attained secondary level education and were full time house wives. Some of them agreed to the assertion that they did not have money for transport or to pay for antenatal care services if they were not free. Low socio-economic status further limits these women to seek antenatal care early as they may not afford the services. This finding is in agreement with other studies [9-14] which have shown that maternal and husband’s education and therefore social-economic status and house hold income affect the utilization of antenatal care.

Although many pregnant women reported a history of spontaneous abortion or a problem in the current or previous pregnancy, coming late for antenatal care implies that they don’t perceive the possibility of the problem (abortion or other complication) reoccurring, which necessitates coming early for antenatal care. Thus many pregnant women don’t recognize the importance of antenatal care, particularly the need to make their first antenatal care visit early in pregnancy. This is in agreement with the findings of similar studies done in South Africa [8,9]. This lack of perception of the importance of antenatal care is also reflected in the pregnant women’s assertion in the results that there is no need to come early for antenatal care if one has no problem with her pregnancy. This also explains why these women gave reasons like “I was busy”, “I was lazy”, “I got tired of attending antenatal care in previous pregnancies”, “I have had children before”. Therefore, there is need to emphasize the importance of early antenatal care during health education for pregnant women.

It is worth noting that 30% of the study participants had sought antenatal care somewhere before coming to Mulago hospital. Besides those who had been referred from other health units (much as they had attended there later than the recommended time for the first visit) or had shifted from one place to another, some simply just wanted to deliver from Mulago hospital and so they wanted to get a file and book for delivery as this is a requirement to deliver from the hospital. This shows lack of confidence by these women in the services provided in the other health units. This suggests need to improve the quality of services being offered in the peripheral heath units so as to gain the pregnant women’s confidence. This will further reduce the problem of multiple antenatal care attendance.

The limitation of this study was that it was conducted in a referral hospital and its participants where only pregnant women who had presented for the first antenatal care visit with a gestation age of more than 20 weeks. Since no information was collected on those who made their first visit earlier than 20 weeks, there is no reference group to verify the reasons for late attendance. The results may have been different in a lower level health facility and if pregnant women who came at a lower gestation age had been included. Therefore, further research is needed to identify reasons for coming early for antenatal care, with inclusion of women who make their first visit before 20 weeks of gestation. The other limitation was that ultrasound scan to confirm gestation age was not performed on all women hence different methods were used to determine gestation age. This may have caused some inaccuracies considering that some women may not have remembered the date of the last normal menstrual period accurately and the estimation by use of ultrasound scan may not be exact especially in the third trimester. However, the results obtained are a fair representation of the timing and reasons for late coming for the first antenatal care visit by pregnant women. These results imply that there is need to reinforce education of communities about the timing and importance of attending antenatal care early.

## Conclusion

In conclusion, this study showed that pregnant women are not well-informed of the right gestation age at which they should make their first antenatal care visit and also of the importance of attending antenatal care early. We therefore recommend that health education on the timing and importance of attending antenatal care early should be done in the communities where women and other people that influence the pregnant woman’s decision to attend antenatal care live so that they get this information even before they conceive. This can be done through the media, places of worship, schools and community gatherings.

## Competing interests

The authors declare that they have no competing interests.

## Authors’ contributions

IK conceived of the study, participated in its design, coordination of the data collection process and draft of the manuscript. DKK and FC participated in the statistical analysis and drafting of the manuscript. SKS, AA, GN and LO participated in the design and coordination of the study. All authors read and approved the final manuscript.

## Pre-publication history

The pre-publication history for this paper can be accessed here:

http://www.biomedcentral.com/1471-2393/13/121/prepub

## References

[B1] WHO Global Health Observatory (GHO) Antenatal care-situations and trends 2011

[B2] Ministry of Health Uganda Clinical Guidelines: antenatal care 2010 325 Revised first edition;

[B3] Weiner EA Billamay S Partridge JC Martinez AM Antenatal Education for Expectant Mothers Results in Sustained Improvement in Knowledge of Newborn Care J Perinatol 2011 31 2 92 97 10.1038/jp.2010.108 20689517

[B4] Dim CC Onah HE The Prevalence of Anemia among Pregnant Women at Booking in Enugu, South Eastern Nigeria Medscape General Medicine 2007 9 3 11 18092018 PMC2100084

[B5] Simkhada B Teijlingen ER Porter M Simkhada P Factors affecting the utilization of antenatal care in developing countries: systematic review of the literature J Adv Nurs 2008 619 3 244 260 18197860 10.1111/j.1365-2648.2007.04532.x

[B6] Uganda Bureau of statistics Uganda demographic and health survey 2011

[B7] Magadi MA Madise NJ Rodrigues RN Frequency and timing of antenatal care in Kenya: explaining the variations between women of different communities Soc Sci Med 2000 51 4 551 561 10.1016/S0277-9536(99)00495-5 10868670

[B8] Myer L Harrison A Why do women seek antenatal care late? Perspectives from rural South Africa J Midwifery Womens Health 2003 48 4 268 272 10.1016/S1526-9523(02)00421-X 12867911

[B9] Phafoli SH Van Aswegen EJ Alberts UU Variables influencing delay in antenatal clinic attendance among teenagers in Lesotho South African Family Practice 2007 49 9

[B10] Karlen S Say L Souza JP Hogue CJ Calles DL Gulmezoglu AM Raine R The Relationship Between Maternal Education and Mortality Among Women Giving Birth in Health Care Institutions BMC Publ Health 2011 11 606 10.1186/1471-2458-11-606 PMC3162526 21801399

[B11] Trinh LT Rubin G Late entry to antenatal care in New South Wales, Australia Reproductive Health 2006 3 8 10.1186/1742-4755-3-8 16916473 PMC1562358

[B12] Ali AA Osman MM Abbaker AO Adam I Use of antenatal care services in Kassala, eastern Sudan BMC Pregnancy Childbirth 2010 10 67 10.1186/1471-2393-10-67 20973972 PMC2987884

[B13] Ye Y Yoshida Y Harun-Or-Rashid M Sakamoto J Factors affecting the utilization of antenatal care services among women in Kham district, Xiengkhouang province, Lao PDR J Med Sci 2010 72 23 33 10.1292/jvms.08-0332 PMC11254371 20229700

[B14] Low P Paterson J Wouldes T Carter S Williams M Percival T Factors affecting antenatal care attendance by mothers of pacific infants living in Newszealand N Z Med J 2005 118 12 16 15937524

